# Extracellular HIV-1 Tat Mediates Increased Glutamate in the CNS Leading to Onset of Senescence and Progression of HAND

**DOI:** 10.3389/fnagi.2020.00168

**Published:** 2020-06-09

**Authors:** Jamie Marino, Brian Wigdahl, Michael R. Nonnemacher

**Affiliations:** ^1^Department of Microbiology and Immunology, Drexel University College of Medicine, Philadelphia, PA, United States; ^2^Center for Molecular Virology and Translational Neuroscience, Institute for Molecular Medicine & Infectious Disease, Drexel University College of Medicine, Philadelphia, PA, United States; ^3^Sidney Kimmel Cancer Center, Thomas Jefferson University, Philadelphia, PA, United States

**Keywords:** HIV-1, Tat, HAND, Alzheimer’s, glutamate, CNS, senescence

## Abstract

Human immunodeficiency virus type 1 (HIV-1)- associated neurocognitive disorders (HAND) is a disease of neurologic impairment that involves mechanisms of damage similar to other degenerative neurologic diseases such as Alzheimer’s disease (AD). In the current era of antiretroviral therapy (ART), HIV-1 replication is well-suppressed, and yet, HIV-1-infected patients still have high levels of chronic inflammation, indicating that factors other than viral replication are contributing to the development of neurocognitive impairment in these patients. The underlying mechanisms of HAND are still unknown, but the HIV-1 protein, Tat, has been highlighted as a potential viral product that contributes to the development of cognitive impairment. In AD, the presence of senescent cells in the CNS has been discussed as a contributing factor to the progression of cognitive decline and may be a mechanism also involved in the development of HAND. This mini-review discusses the viral protein HIV-1 Tat, and its potential to induce senescence in the CNS, contributing to the development of HAND.

## Introduction

Human immunodeficiency virus type 1 (HIV-1)-associated neurocognitive disorders (HAND) represents a range of neurologic impairments observed in HIV-1-infected patients who are well-suppressed on antiretroviral therapy (ART) (Dickens et al., 2017; Williams et al., 2020). HAND is diagnosed by neurocognitive testing and is further classified based on the level of impairment that interferes with activities of daily living, where the most common clinical characteristics are subcortical dementia with deficits in memory, attention, and concentration (Nightingale et al., 2014; Eggers et al., 2017). Although implementation of ART has decreased the severity of HAND, it still remains prevalent, with over 50% of HIV-1-infected patients exhibiting mild symptoms of HAND (Chai et al., 2017). Because HAND remains prevalent even in the ART era, it has been suggested that viral replication is not a major factor involved in mediating cognitive decline (Zeitler et al., 2015; Berman et al., 2016; Dickens et al., 2017; Cohen et al., 2018). The viral protein HIV-1 Tat therefore, has been highlighted as a potential viral product that may contribute to the development of HAND (Chen et al., 1997; Eugenin et al., 2003). Tat is an early gene product of HIV-1, and is continually secreted from latently infected cells (Mele et al., 2018; Link et al., 2019; Spector et al., 2019), where extracellular Tat has been detected in 36.8% of patient CSF samples at concentrations ranging from 200 pg/ml to 6.5 ng/ml (Henderson et al., 2019). Extracellular Tat is biologically active and can cause induction of apoptosis, oxidative stress, and release of pro-inflammatory cytokines and neurotransmitters, all of which may contribute to the development of HAND (Table 1; Henderson et al., 2019; Ajasin and Eugenin, 2020). Additionally, the presence of Tat alone in the CNS of animal models is sufficient to replicate HAND pathologic and behavioral changes (Zeitler et al., 2015; Berman et al., 2016; Dickens et al., 2017; Cohen et al., 2018).

**TABLE 1:** Observed changes in senescent cells and in Tat-exposed cells.

Senescence-induced changes	Tat-induced changes	References
Senescent cells have disorganized and diffuse TJP expression and increased BBB permeability	Tat exposure in astrocytes results in increased MMP-9 and cleaved occludin. BMECs exposed to Tat have decreased occludin and claudin-5 TJP mRNA	Johnston et al., 2001^T^; Andras et al., 2005^T^; Eugenin et al., 2011^T^; Rao et al., 2014^T^; Atluri et al., 2015^T^; Yamazaki et al., 2016^S^
Senescent cells secrete pro-inflammatory cytokines including Increased secretion of IL-6, CCL5, IL-8, MMP-1	BMECs exposed to Tat secrete IL-6 and TNF-α. Tat activates transcription factors like Sp1 and NF-κB to increase production of pro-inflammatory cytokines TNF-α, CCL2, IL-2, IL-6, CCL5, and IL-8. Neurons exposed to Tat produce TNF-α, and IL-1β	Liu et al., 2011^S^; Rao et al., 2014^T^; Dickens et al., 2017^*B*^; Baker and Petersen, 2018^S^; Cohen et al., 2018^S^; Herranz and Gil, 2018^S^; Hijmans et al., 2018^S^; Ajasin and Eugenin, 2020^T^
Increased expression of ICAM-1	Tat alters the expression of adhesion molecules such as ZO-1, JAM-A, PECAM-1 and CD99	Bhat et al., 2012^S^; Baker and Petersen, 2018^S^; Ajasin and Eugenin, 2020^T^
Increased oxidative stress (including ROS and NO production)	Tat exposure can increase oxidative stress (can increase ROS and NO production by causing ER stress)	Capone et al., 2013^T^; Baker and Petersen, 2018^S^; Jeon et al., 2018^S^
Increased expression of p16 and p21	Tat exposure on MSCs resulted in increased p21 expression	Liu et al., 2011^S^; Bhat et al., 2012^S^; Beaupere et al., 2015^S,T^; Baker and Petersen, 2018^S^; Cohen et al., 2018^S^
A feature of senescence is the permanent exit and the cell cycle and block in cell growth	Tat exposure reduced population doubling in MSCs, and reduced cell growth in U373. MG cells, primary mouse astrocytes and 293T cells	Zhou et al., 2004^T^; Beaupere et al., 2015^S,T^; Childs et al., 2018^S^; Langford et al., 2018^T^

*^*S*^, references for senescence-induced changes; ^*T*^, references for Tat induced changes.*

A major mechanism of HIV neuropathogenesis involves infiltration of immune cells, such as lymphocytes and macrophages, across the blood-brain barrier (BBB), and into the CNS (Rao et al., 2014; Eggers et al., 2017). These cells can then produce chemokines and cytokines, such as MCP-1 and TNF-α, to recruit more cells from the periphery into the CNS, while also creating a pro-inflammatory environment within the CNS (Rao et al., 2014; Eggers et al., 2017). Infected immune cells within the CNS can then infect CNS resident microglia and possibly astrocytes, and newly infected cells will continually secrete viral proteins and contribute to inflammation (Andras et al., 2005).

Although neurons cannot become infected by HIV-1, they can become damaged by viral proteins, such as Tat, inflammatory cytokines, such as TNF-α and IL-1β, and small metabolites, such as reactive oxygen species (ROS) and nitric oxide (NO) (Rao et al., 2014). The resulting neuronal damage can lead to apoptosis and loss of dendrite processes where these changes have been shown to contribute to HAND development (Rao et al., 2014).

## Hand and Alzheimer’s Disease

A potentially significant contributor of HAND pathology is beta-amyloid (Aβ), where Aβ deposition is accelerated in HIV-1 patients as compared to uninfected, age-matched controls (Clifford et al., 2009; Chai et al., 2017; Eggers et al., 2017). Accumulation of amyloid plaques is associated with neurodegenerative conditions such as Alzheimer’s Disease (AD) and amyloid measurements in the CSF of HAND patients has been reported to be similar to those in Alzheimer’s type dementia (Clifford et al., 2009; Chai et al., 2017). However, there are distinct differences in Aβ deposition in AD as compared to HAND, where HAND patients have diffuse amyloid plaques, while AD patients have senile plaques and neurofibrillary tangles (Clifford et al., 2009; Canet et al., 2018). Chai et al. (2017) HIV-1 Tat can also mediate amyloid dysfunction as Tat can inhibit neprolysin, which is an endonuclease necessary for Aβ breakdown (Clifford et al., 2009). Tat can also compete with amyloid precursor protein (APP) and apolipoproteins E (an Aβ chaperone), for binding to the low-density lipoprotein receptor related protein (LRP), and this will result in a blockage of LRP-mediated clearance of Aβ from the brain (Clifford et al., 2009). Similar to AD, patients with HAND also have significantly decreased levels of Aβ42, which is the cleavage product of APP, while tau protein is increased, suggesting that HAND may be associated with AD-like processes (Clifford et al., 2009; Eggers et al., 2017).

Both AD and HAND pathologies result in neurodegeneration and cognitive impairment that progresses over time. As discussed previously, HAND is characterized by immune cell infiltrate into the CNS and production of pro-inflammatory cytokines (Rao et al., 2014; Eggers et al., 2017). Similar changes have also been detected in AD, where studies have reported chronic CNS inflammation (increased IL-1β and IL-6), as well as increased immune cell infiltrate in the CNS (Eugenin et al., 2011; Bhat et al., 2012; Atluri et al., 2015; Chen et al., 2017; Dickens et al., 2017; Baker and Petersen, 2018; Madeira et al., 2018). The mechanisms involved in neurodegenerative diseases such as AD and HAND, although not identical, are similar and have some overlapping features. Because some similarities in underlying mechanisms of AD and HAND have already been identified, it is possible that there are additional similarities, yet to be reported. One such pathway is senescence, which has been discussed in AD as a mechanism that both contributes to and results from cell stress and inflammation. Due to the similarities between HAND and AD, we propose a possible HIV-1-Tat-induced mechanism of senescence in the CNS of HIV patients that exhibit HAND.

## Senescence

Senescence is a cell mechanism usually involved in aging that is driven by telomere shortening or oxidative stress and results in a permanent exit of the cell cycle, and resistance to apoptosis (Childs et al., 2018). When senescence occurs prematurely, it is associated with neurodegenerative disorders such as AD, and it is possible that premature CNS senescence could also be an underlying mechanism in the development of HAND (Cohen et al., 2018). Premature senescence can occur in human astrocytes when exposed to high levels of oxidative stress, and involves increased p16^ink4a^ and p21, decreased telomere length, and increased secretion of IL-6, CCL5, IL-8 and intercellular adhesion molecule 1 (I-CAM-1) (Bhat et al., 2012; Baker and Petersen, 2018). Astrocytes also undergo senescence in response to ROS, and in astrocytes derived from brains of AD patients, there was increased expression of the cell cycle regulator p16, as well as increased matrix metallopeptidase-1 (MMP-1) and IL-6, all of which have also been shown to be upregulated in senescence (Baker and Petersen, 2018).

As previously discussed, Tat can mediate oxidative stress and inflammation, both of which are driving factors in the development of premature senescence (Table 1; Capone et al., 2013; Baker and Petersen, 2018; Jeon et al., 2018). Although Tat can be produced and secreted by infected CNS cells, peripherally produced Tat can also cross the blood-brain barrier (BBB), and interact with and damage cells of the barrier (Buscemi et al., 2007; Debaisieux et al., 2012; Ma et al., 2016). The BBB is composed of brain microvascular endothelial cells (BMECs) and astrocytes and acts as a semi-permeable barrier that restricts entry into the CNS, a function that is essential for proper brain function (Kanmogne et al., 2007; Eugenin et al., 2011; Strazza et al., 2011; Williams et al., 2012; Atluri et al., 2015; Yamazaki et al., 2016). Endothelial cells at the BBB are surrounded by pericytes that help promote endothelial cell survival and contribute to stabilization of capillaries (Nakagawa et al., 2012). Dysregulation of the BBB, caused by HIV-1 can exacerbate neurodegenerative disorders by allowing aberrant influx of cells into the CNS, leading to increased inflammation and neurotoxicity (Rao et al., 2014; Yamazaki et al., 2016; Baker and Petersen, 2018).

In mesenchymal stem cells (MSCs), a precursor to astrocytes, extracellular Tat reduced population doublings and increase p21 expression, both indicators of senescence (Beaupere et al., 2015). Furthermore, senescent astrocytes lose their neuroprotective function, and can negatively impact surrounding cells and tissues primarily through production of a pro-inflammatory cytokines (Table 1; Kanmogne et al., 2007; Liu et al., 2011; Williams et al., 2012; Yamazaki et al., 2016; Cohen et al., 2018; Herranz and Gil, 2018; Jeon et al., 2018). This is important because astrocytes exposed to Tat produce pro-inflammatory cytokines, and this may indicate the ability of Tat exposure to induce senescence in astrocytes.

Although BMECs at the BBB cannot be infected by HIV-1, they are significantly affected by the indirect action of HIV-1, such as through the activity of viral proteins like Tat, where BMECs may undergo senescence in response to external stressors (Johnston et al., 2001; Liu et al., 2011; Williams et al., 2012; Rahimian and He, 2016; Cohen et al., 2018; Herranz and Gil, 2018; Jeon et al., 2018). In BMECs treated with Tat at 500 ng/ml for 24 h, there was increased senescence and production of IL-6 and TNF-α (Table 1; Hijmans et al., 2018). Senescence can also affect TJPs, and in a co-culture system of senescent murine BBB cells, the TJP expression level was unchanged, however, structurally, the TJP were disorganized and diffuse, and resulted in increased BBB permeability as measured by Evans Blue (Table 1; Yamazaki et al., 2016). Decreased BBB integrity occurs in HIV-1 infection, where the loss of BBB integrity, potentially due to exposure to Tat and altered glutamate levels may induce the onset of senescence in astrocytes and BMECs, the major cells of the BBB, culminating in the generation of neurocognitive disorders such as HAND (Beaupere et al., 2015; Yamazaki et al., 2016; Hijmans et al., 2018).

## Glutamate in CNS

Glutamate is the major excitatory neurotransmitter in the CNS, and is tightly regulated where high levels of glutamate will lead to excitotoxicity and neuronal death (Andras et al., 2007; Madeira et al., 2018). Na+-dependent excitatory amino acid transporters (EAATs) are responsible for the removal of extracellular glutamate in the CNS, and regulate extracellular concentrations of glutamate (Magi et al., 2019). The glial glutamate transporter, EAAT-2 is located on astrocytes and is the major transporter responsible for glutamate uptake (Erdo et al., 2017). Tat can negatively affect EAAT-2 functioning, and in a study of primary cultured mouse astrocytes treated with recombinant Tat for 48 h there was decreased EAAT-2 protein and mRNA expression, resulting in high levels of extracellular glutamate (Ye et al., 2017). Additionally, in neuroblastoma cells treated with extracellular subtype B Tat, there was significantly increased glutamate as compared to subtype C Tat-treated cells (Williams et al., 2020). This observation is important because subtype B Tat has been linked to increased neurotoxicity as compared to subtype C Tat, indicating that altered glutamate levels may be a major factor responsible for neurotoxicity (Williams et al., 2020). Furthermore, astrocytes exposed to subtype B Tat had a reduced capacity for buffering glutamate, as well as impaired glutamate intake as compared to subtype C Tat, resulting in higher concentrations of extracellular glutamate with subtype B Tat (Zhou et al., 2004; Zou et al., 2011).

Tat may also simultaneously increase extracellular levels of glutamate in the CNS by binding to Connexin 43 (Ye et al., 2017). Connexin 43 is a gap junction protein that connects the cytoplasmic compartments of coupled cells, which allows the cells to directly interact with one another (Berman et al., 2016). Connexin 43 controls the release of glutamate from astrocytes into the extracellular space, where astrocytes are a major cell responsible for maintaining glutamate homeostasis (He et al., 2020). Importantly, Tat is the only HIV-1 protein capable of interacting with and significantly increasing the expression of Connexin 43 on astrocytes (Berman et al., 2016). Efflux of glutamate from astrocytes due to Connexin 43 activation leads to increased glutamate in the extracellular space, which can result in neuronal excitotoxicity and death (He et al., 2020). Importantly, Connexin 43 has a key role in learning and memory and when normal functions of Connexin 43 were interrupted it led to impaired hippocampal spatial memory (He et al., 2020). Increased expression of Connexin 43 has also been detected in AD, where Connexin 43 was specifically increased at amyloid plaques (He et al., 2020). The development of AD symptoms has also been linked to increased glutamate resulting from activation of Connexin 43 on astrocytes (He et al., 2020). Increased Connexin 43 in astrocytes will result in increased calcium levels, which leads to the development of ER stress and can culminate in neuronal damage and cognitive decline, in both HAND and AD (He et al., 2020). The ability of Tat to alter the regulatory mechanisms of glutamate results in increased concentrations of extracellular glutamate and can have significant consequences on normal CNS signaling pathways (Magi et al., 2019).

## Changes in Signaling Pathways

In normal signaling pathways in the CNS, extracellular glutamate will interact with and activate synaptic *N-*methyl-D-aspartate receptors (NMDARs), resulting in neuroprotection (Andras et al., 2007; Zhou and Sheng, 2013; Magi et al., 2019). NMDARs are a subclass of postsynaptic neuronal glutamate receptors that mediate cellular calcium entry and are involved in cell signaling (Zhou and Sheng, 2013; Madeira et al., 2018). Activation of NMDARs requires simultaneous binding of both glutamate and glycine/D-serine to the GluN2 and GluN1 subunits of NMDAR (Vieira et al., 2020). In a normal physiologic system, glutamate is removed from the synaptic cleft and converted into glutamine by glutamine synthetase, where glutamine is then transported back and converted to glutamate by glutaminase (Madeira et al., 2018). However, excitotoxicity caused by excessive glutamate release from the presynaptic terminal can lead to NMDAR hyperactivation and result in excitotoxicity, which contributes to neurological degeneration (Vieira et al., 2020). Excessive extracellular glutamate can bind to NMDARs located in the extra-synaptic regions of neurons and lead to neurotoxicity (Zhou and Sheng, 2013). It has also been shown that Aβ can stimulate extra-synaptic NMDARs to increase the neuronal production of Aβ to further contribute to CNS dysfunction (Zhou and Sheng, 2013).

Tat is able to stimulate NMDARs, and Tat-mediated stimulation can lead to glutamate-dependent excitotoxicity and apoptosis in neurons (Haughey et al., 2001; Sharp et al., 2003; Hogan-Cann and Anderson, 2016; Ye et al., 2017). Additionally, excitotoxicity resulting from Tat exposure is enhanced by the addition of glutamate, which results in calcium flux from neurons where excessive calcium may be involved in neuroinflammation (Haughey et al., 2001; Hogan-Cann and Anderson, 2016). Extracellular Tat can also potentiate excitotoxicity to lead to neuronal cell death by increasing the phosphorylation of NMDAR subunits in rat cortical neurons (Haughey et al., 2001). Additionally, over-activation of NMDARs has been previously linked to the development of AD, where there are increased levels of both glutamate and glutamine in the CSF, as compared to healthy controls, and patients with higher glutamate and glutamine levels also had lower neurocognitive testing scores (Madeira et al., 2018).

Glutamate excitotoxicity can also lead to a cascade of inflammation in the CNS, where glutamate can increase the levels of nitric oxide synthase (NOS), which has been linked to CNS dysfunction (Madeira et al., 2018; Williams et al., 2020). At the BBB, exposure of BMECs to high concentrations of glutamate resulted in the formation of ROS leading to oxidative stress, as well as decreased *trans*-endothelial electrical resistance (TEER) and re-localization of occludin, a major tight junction protein (TJP) at the BBB, suggesting that high concentrations of extracellular glutamate can negatively affect BBB integrity (Capone et al., 2013).

Although cytokines and ROS each have roles in CNS damage during HIV-1 infection, alterations of TJPs has been shown to be the most predominant mechanism underlying BBB disruption (Almutairi et al., 2016). In astrocytes, Tat exposure led to increased MMP-9 which disrupted the BBB by cleaving occludin to inactivate it (Johnston et al., 2001; Rao et al., 2014; Atluri et al., 2015). Similarly, BMECs exposed to Tat had decreased occludin and claudin-5 TJP mRNA (Andras et al., 2005; Atluri et al., 2015). Redistribution and inhibition of TJPs due to Tat exposure resulting in increased glutamate concentrations will result in impaired BBB integrity, which can allow cells and protein to more easily cross the BBB to enter into the CNS to lead to damage and inflammation.

Tat exposure has also been shown to lead to oxidative stress and can induce transcription pathways like NF-κB and AP-1, to produce the pro-inflammatory cytokines IL-6 and IL-8 in BMECs (Li et al., 2009; Rizzo and Leaver, 2010; Labus et al., 2014; Youn et al., 2017). Astrocytes exposed to Tat had increased NF-κB, resulting in the production of MCP-1, IL-1β, IL-6, IL-8, chemokine ligand 5 (CCL5/RANTES), interferon-γ-induced protein-10 (IP-10/CXCL10), and tumor necrosis factor alpha (TNF-α) (Table 1; Boven et al., 1999; Weiss et al., 1999; Nookala and Kumar, 2014; Almutairi et al., 2016; Fan and He, 2016). Alteration of signaling pathways in the CNS, either by direct Tat interactions, or indirectly, through Tat-altered glutamate signaling, can result in increased cell stress and damage, and together these changes will culminate in the onset of senescence in the CNS.

## Discussion

**FIGURE 1:**
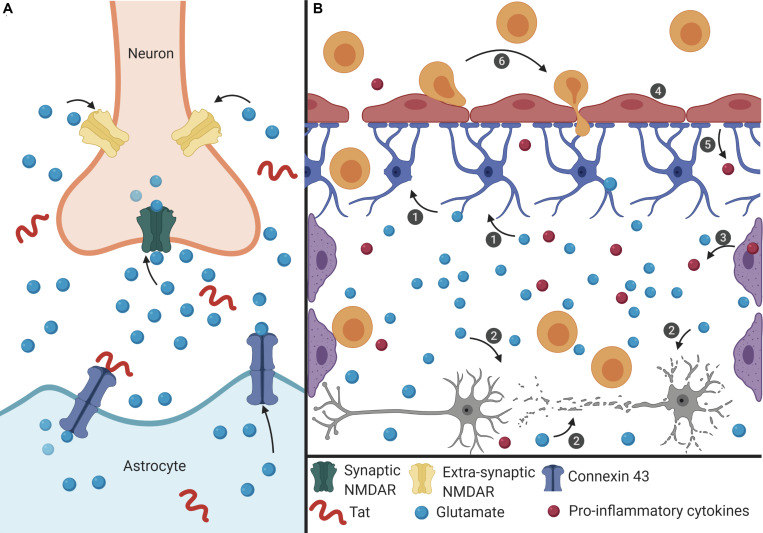
Proposed mechanism of Tat-induced senescence. **(A)** Extracellular Tat binds to Connexin 43 and decreases EAAT-2 expression on astrocytes to result in increased extracellular glutamate concentrations. Under normal conditions, glutamate binds to synaptic NMDARs (green) to promote neuroprotective pathways. However, high concentrations of extracellular glutamate will allow glutamate to exit the synaptic cleft and bind to extra-synaptic NMDARs (pink), which promotes excitotoxicity. **(B)** The interaction depicted in panel **(A)** has widespread effects on other cells of the CNS. (1) Extracellular glutamate causes cell stress and damage in astrocytes (blue), depicted as loss of foot process, and (2) neurons (gray), depicted as synaptodendritic damage, and (3) causes stress in perivascular macrophages (purple), depicted as an increase in proinflammatory cytokines. Extracellular glutamate also affects (4) endothelial cells (red), causing increased permeability, (5) and release of ROS and pro-inflammatory cytokines (red), which results in (6) recruitment of immune cells (orange) across the BBB and into the CNS, creating a feedback loop of chronic inflammation, and damage. This inflammatory microenvironment will force cells to become senescent, and this will lead to further decreased BBB integrity and production of pro-inflammatory cytokines.

Based on the literature, Tat-induced senescence represents a possible mechanism underlying the development of HAND (Figure 1). Here we link data from multiple studies to discuss a potential mechanism of Tat-induced senescence.

Extracellular Tat can interact with Connexin 43 on astrocytes, which is involved in glutamate regulation. Tat binding to Connexin 43 opens the channel, resulting in efflux of glutamate from the astrocytes and into the extracellular environment (Berman et al., 2016; Figure 1A). At the same time, Tat can also decrease the expression of EAAT-2 on astrocytes, which in turn decreases glutamate uptake (Ye et al., 2017). Together, these two effects on astrocytes will lead to high concentrations of extracellular glutamate in the CNS. As glutamate is a highly regulated neurotransmitter in the CNS, cells are sensitive to alterations in the concentration of glutamate (Madeira et al., 2018). The ability of Tat to alter two of the major mechanisms used to regulate the level of glutamate will have significant effects on other cells of the CNS.

High levels of extracellular glutamate will allow it to bind to extra-synaptic NMDARs, rather than the normal synaptic NMDARs, and this extra-synaptic activation will potentiate excitotoxicity (Zhou and Sheng, 2013; Madeira et al., 2018). In other cells of the CNS, such as perivascular macrophages and BMECs, glutamate can cause cell stress, leading to the release of pro-inflammatory cytokines and ROS (He et al., 2020; Williams et al., 2020). These effects culminate in a chronic feedback loop where the pro-inflammatory environment creates additional cell stress and oxidative damage, and this leads to immune infiltrate across the BBB and into the CNS (Figure 1B). As a result, there is chronic inflammation and damage in the CNS which can cause neuronal death, and in response to the inflammatory extracellular environment, cells of the CNS, such as astrocytes and BMECs, become senescent in order to avoid further cell stress and damage that would lead to apoptosis. Additionally, in HAND there is a loss of pericytes at the BBB, which are important to promote endothelial cell survival (Niu et al., 2015). Onset of senescence further reinforces a pro-inflammatory feedback loop to promote an inflammatory microenvironment that can further induce adjacent cells to also become senescent. As cells at the BBB become senescent, there is redistribution of tight junction proteins and changes in cell morphology that affects the integrity of the barrier, thus enabling more cells to cross into the CNS (Yamazaki et al., 2016). Establishment of this inflammatory feedback mechanism will cause multiple cells of the CNS to become senescent which can result in neurocognitive impairment and lead to cognitive decline.

Tat is a small HIV-1 protein that is capable of causing large scale damage due to its ability to alter multiple cellular pathways, including glutamate regulation in the CNS. Senescence of CNS cells has been identified as an underlying mechanism responsible for cognitive impairment in AD, a condition that shares many mechanistic similarities to other neurodegenerative disorders, such as HAND (Table 1). Further studies elucidating the underlying mechanisms of Tat-induced dysregulation will be important to understand how these interactions may influence the development of HAND. Additionally, determining the impact of Tat-induced senescence in the CNS is important to understand the contribution of senescence to the pathogenesis of HAND (Yamazaki et al., 2016; Baker and Petersen, 2018).

Although this mini-review has focused on Tat and its impact on glutamate levels and induction of senescence, there are other HIV-1 proteins that are relevant to the development of CNS dysfunction. The viral proteins Nef and Gag are released from HIV-1-infected cells via exosomes and Nef can cause activation-induced cell death and cause neuronal toxicity (Hu et al., 2016; Dickens et al., 2017). Apoptosis has been observed in BMECs at the BBB exposed to gp120, Vpr, or Nef which has indicated the ability of these viral proteins to alter BBB integrity (Toborek et al., 2005). Additionally, exposure of astrocytes to extracellular Vpr led to increased concentrations of IL-6, IL-8, and MCP-1 as well as increased production of ROS due to an increase in the level of oxidized glutathione (GSSG), and these effects may result in senescence onset (Ferrucci et al., 2012, 2013). Finally, a study examining Tat and Nef showed that exposure of human mesenchymal stem cells to either protein had increased senescence after 20 days, indicating the ability of other HIV-1 proteins to induce senescence in non-CNS cell types (Beaupere et al., 2015). While multiple HIV-1 proteins can mediate effects similar to Tat in the CNS, it has been suggested that examining combination effects is warranted. However, it is important to keep in mind that Tat seems to be the one protein still measurable in the CNS during ART.

## Author Contributions

JM and MN conceived and designed the study. All authors prepared and designed the figures, drafted the manuscript, and read and approved the final manuscript.

## Conflict of Interest

The authors declare that the research was conducted in the absence of any commercial or financial relationships that could be construed as a potential conflict of interest.
